# Digital Learning As Enhanced Learning Processing? Cognitive Evidence for New insight of Smart Learning

**DOI:** 10.3389/fpsyg.2017.01329

**Published:** 2017-08-03

**Authors:** Dina Di Giacomo, Jessica Ranieri, Pilar Lacasa

**Affiliations:** ^1^Department of Life, Health and Sciences, University of L’Aquila L’Aquila, Italy; ^2^Department of Psychology, Documentation and Audiovisual Communication, University of Alcalá Alcalá de Henares, Spain

**Keywords:** digital skills, cognitive process, childhood, technology, enhancing of cognitive processing

## Abstract

Large use of technology improved quality of life across aging and favoring the development of digital skills. Digital skills can be considered an enhancing to human cognitive activities. New research trend is about the impact of the technology in the elaboration information processing of the children. We wanted to analyze the influence of technology in early age evaluating the impact on cognition. We investigated the performance of a sample composed of n. 191 children in school age distributed in two groups as users: high digital users and low digital users. We measured the verbal and visuoperceptual cognitive performance of children by n. 8 standardized psychological tests and *ad hoc* self-report questionnaire. Results have evidenced the influence of digital exposition on cognitive development: the cognitive performance is looked enhanced and better developed: high digital users performed better in naming, semantic, visual memory and logical reasoning tasks. Our finding confirms the data present in literature and suggests the strong impact of the technology using not only in the social, educational and quality of life of the people, but also it outlines the functionality and the effect of the digital exposition in early age; increased cognitive abilities of the children tailor digital skilled generation with enhanced cognitive processing toward to smart learning.

## Introduction

The influence of technology on people life is almost evident. The impact of technology is on the quality of life and wellness such as better living in social context, improved efficiency in working context and enhanced educational system. Lately, technology have had large and quick spread out making better: (a) the management of the working performance reducing elaboration processing data and providing fast processing of tasks, (b) the healthcare efficacy by application of the technology in the screening, diagnosis and treatment of the patients, (c) the educational proficiency enhancing the learning processing in lifespan in order to favor equality and wellness in daily living. Childhood, adulthood, and old age have taken advantage by technology through national and international scientific synergies and investments, providing innovative solutions for people’s needs (Tikhomirov, 1974; Gamberini et al., 2006; Githens, 2007; Blaschke et al., 2009; Wang et al., 2010).

‘Digital native’ or ‘Net generation’ are ones of most used labels applied to the young people defining the generation of ‘tech-savvy’ learners (Tapscott, 1998; Prensky, 2001). Cognitive, social and emotional development processing results influenced by technology; as well as the related educational practice seems reflecting a changing in the daily living.

Interesting research topic is the influence of young children’s use of technology on the learning system of young generation. Initially, researchers were rather cautious and they have been oriented to highlight the technology as impediment for mental development. In short time, the research addressed opposite view. Armstrong and Casement (2000) pointed out the technology should be avoided in order to don’t keep out of the reach of children from interaction with others and so do not influence negatively the social and emotional development. Following review studies have evidenced that the concept about the technology as impediment in mental development as to be a myth to debunk (Yelland, 2011; Plowman and McPake, 2013): the awarded use of technology should be considered a valid instrument to improve the growth of children and doesn’t represent an impediment for mental development.

Particular lively debate is about the technological impact on the educational system promoting new kind of learners. In fact, the radical occurred changing in education is strictly joined to the rapid technological development providing an impressive gap between the traditional teaching method and the needs of young people (Bennett and Maton, 2010). Moreover, Hsin et al. (2014) have highlighted the presence of fundamental gap between the technologically skilled and technological unskilled individuals, and the influent factors can be: economical status, cultural and social context, parents education. Kerawalla and Crook (2002) has pointed out as the home access to the technology can be strongly measure of the technological advantage: costs of devices affect the exposition to the digital tools and so having a low effect on the development of digital skill. Location of computer, rules about access and value placed on technology are three main determinant factors in the home access to the device that can mark the difference among young generation (Facer and Furlong, 2001; Campbell et al., 2006): timing, exposition time, availability of experienced digital tools can be somehow the measures of technological skill development. Besides, Furlong et al. (2000) suggested that the availability of computer doesn’t mean genuine access. Parental control and much more parental supervising and supporting are getting became the focal key point in the early stage of digital skills.

The suggestion of these studies addressed that the new generations grow living inhomogeneous exposition to the technology (in terms of access to the device and digital tools use) and that draw different opportunities so then different technology experience that makes the difference the development of digital skills. Bennett and Maton (2010) sustained that the young people are pushed to the diversity of interests, motivations and needs. The simple exposition to the technology doesn’t mean to be digital (or native) skilled. The complexity of young people as technological skilled has to be undertaken better and it is getting became the emerging research agenda. Specific focus has to be reserved to the impact of the technology on the intellective development in lifelong learning: as daily evident, the digital skills represent the enhancing of learning processing but those are detected by qualitative data (see review Hsin et al., 2014): it misses quantitative elaboration data about the use of technology influence starting in young age and then in lifelong going.

Verenikina et al. (2003) have evidenced the understanding of the digital exposition like new research trend in order to contribute to analyze the influence of its on cognitive and mental development in childhood. The utility and effectiveness of the technologies in childhood are largely spread out, but literature is focused rather on external variables as measurement of their influence: for instance scholastic successful and/or gaming ability. However, even though the scholastic successful could be a good measure, it doesn’t explain if digital capabilities have effect on intellective improvement of children. In other hand, gaming and pedagogical supports are two relevant areas of Information Technology System and users. Measures about the influence of the exposition to technology in early age can define better the impact of its on intellective development in order to better understand and manage its application into educational system. According to Griffiths (2002), the gaming gets educational benefits not only in terms of entertainment value but also in the increasing of skills. Indeed, Author showed the efficacy of the technology in the development of skills among special need groups: e.g., brain-wave biofeedback on children with attention deficit disorders, or in general in rehabilitation in which several case reports to stimulate the motivation; enlarge the investigation from vulnerable to more largely population could favor the better use of technology based on awareness of its usefully in the enhanced growth of youth. In particular, Cecilia et al. (2015) have analyzed the influence of gaming activities on cognitive performance. The results showed the technological exposition in childhood can favor a better cognitive flexibility and enhanced learning; the autonomy in the use of the technological tools and/or applications represents a good practice to improve the learning abilities in developmental age.

Hsin et al. (2014) have analyzed a review about the influence of technology on learning processes of young children. The interaction children-technology is driven by different modality; the children characteristics are distinguished in age, experience, time and gender; the characteristics of technologies are located in mechanism design, teaching and learning approaches applied and content of technology. This complex interaction is moderated by third factor: adult influence articulated in facilitating children’s engagement in technology, adapting teaching to integrate technology, perceptions of technology, adults’ interaction maximizes the effect of technology. Hsin et al. (2014) proposed a conceptualizing young children’s learning with technology highlighting the factors related to the learning outcomes. In this regard, interesting is the topic about the digital impact in weak leaning processing during childhood. Even if increasing slowly, few studies have been conducted so far. Cofini et al. (2012) have conducted a study about the effectiveness of digital support in poor comprehender children. Starting from innovative approach, experimental digital tool based on Adaptive Learning System (ALS), Authors provided a digital tool in order to support the cognitive weakness of children in reading tasks. Findings showed positive impact on reading performance of poor comprehenders evidencing an added value of technology in learning processing.

In addition, Di Giacomo et al. (2015) have highlighted an increasing performance in the lower reading children applying a smarty digital tool. The results suggested the efficacy and the positive influence of technologies in the cognitive process: in the silent reading, the child may be better stimulated to learn and to comprehend the information using technology interactive. The adaptive learning technology might be considered a strong ally in mental development.

Albeit progressively, scientific community is focusing more deeply on ‘how much’ and ‘what about’ the technology can influence the intellective growth of children. Some studies have been conducted analyzing the impact of the technology in childhood evaluating the school performance (scholar successful data), others are have examined the gaming performance (Di Giacomo et al., 2016; Gomez et al., 2016; Monjelat et al., 2016). These investigations are focused on the performing data on specific digital tasks by processing the behavioral data. Being explored yet is the impact of digital stimulation on cognitive performance in mental development: particularly, evaluating the cognitive performance would mean to measure the digital impact on intellective development, and might be more suitable to argument about new insight of smart learning, no more in the view of digital and cognition distinguished.

Aim of present study was to investigate the influence of digital experience on cognitive development in childhood by evidence-based approach; our objective was to detect if the access to digital gaming in early childhood could determine a changing into mental development. We wanted to measure the effect of technology influence on childhood by assessment of cognitive performance using standardized psychological tests. Our research wants to detect preliminary data in order to understand if the digital experience in early age (or later) impacts the cognition.

## Materials and Methods

### Subjects

The sample was composed of n. 191 native Italian speaker children (n. 82 females; n. 109 males), in range age 7–10 (mean age 8.7 ± 1.1).

The recruitment has been in a public primary Italian school and was mandatory the signed Informed Consensus by parents to process the inclusion of the children.

The inclusion criteria to take part of the sample have been: (a) range age 7–10 years olds, (b) no sign of psychological weakness and/or behavioral deficits, (c) signed Informed consent, (d) filled in of Self-report Questionnaire by parents. The exclusion criteria have been: (a) the presence of neurological and/or psychiatric signs; (b) no signed Informed consent, (c) ambiguity or refusal to fill in the Self-report Questionnaire by parents.

In **Table 1** are reported the details of demographic data of sample.

**Table 1:** Demographic data of the sample.

	Age		Father age		Mother age		Educational years of father		Educational years of mother	
	*M*	*SD*	*M*	*SD*	*M*	*SD*	*M*	*SD*	*M*	*SD*
Male (n. 109)	8.8	1.1	45.3	4.3	40.8	4.6	12.2	3.6	13.3	3.4
Female (n. 82)	8.5	1.1	43.1	5.5	39.2	5.0	12.6	3.6	12.9	3.6
Total (n. 191)	8.7	1.1	44.4	4.9	40.1	4.6	12.4	3.6	13.1	3.5

### Test

To test the cognitive performance of children we applied a Psychological measurement (psychological test composed of n. 8 standardized cognitive tests) and the Self-report Questionnaire was carried out *ad hoc* to detect the technology use from children.

#### Psychological Measurement

The psychological measurement was conducted applying the psychological battery composed of eight standardized cognitive tests divided in two functional testing: visuospatial and verbal task. Visuospatial tasks are: Tower of London, Attentional Matrices, Corsi Blocking Tapping Test, Raven Progressive Matrices. Verbal tasks are: Fluency, Phonological Fluency, Syntactic Comprehension, Naming.

– Tower of London test (Bisiacchi et al., 2005) assesses the executive functioning to detect the planning ability. The tests consist of two boards with pegs and several beads with different colors. The examiner uses the beads and the boards to present the child with problem solving tasks.
– Attentional Matrices test (Bisiacchi et al., 2005) measures sustained attention. The test consists of three matrices of numbers and targets to distinguish. Speed and accuracy performance are detected.
– Corsi Blocking Tapping Test (Bisiacchi et al., 2005) evaluates the visual memory ability. The test consists of nine blocks positioned on a plane. The examiner touches someone of them by fixed sequence and the child has to repeat the same touching sequence. The span of sequence increases progressively.
– Raven Progressive Colored Matrices (Raven, 1949) estimates the logical visual reasoning. The test consists of a colored target missing a piece and six choices: the child has to indicate the piece fits correctly with target.
– Fluency (Bisiacchi et al., 2005) measures the ability to recover words by category criteria. The examiner asks to name words by specific semantic categories in time of 1 min.
– Phonological Fluency (Bisiacchi et al., 2005) evaluates the ability of recover the words by lexical criteria: the examiner ask to list the words starting by specific letter of alphabet, in 1 min.
– Syntactic Comprehension (Bisiacchi et al., 2005) measures the syntactic ability by the comprehension of meaning of sentences. The test consists of drawn items and the child has to indicate the object/action listened by examiner.
– Naming (Bisiacchi et al., 2005) measures the ability to say the name of frequent objects. The test consists of draws of objects and the child has to name of object.

The psychological battery is composed of standardized tests and in the scoring was applied the standard foreseen procedures.

#### Self-Report Questionnaire

A Self-report Questionnaire was carried out to detect information about the intensity of daily use of digital/board games of the sample. The examined variables have been: (a) the using of digital games (PC/laptop, Ipad, Wii/Xbox, PSP, Smartphone, others); (b) the daily time in the using of digital games (half hour, 1 hour, 2–3 hour, more than 3 hours); (c) the using of board games (Monopoli, Risiko, Chess, Lady’s play, Domino, Scarabeo, Goose game, Puzzle, Card’s play, Other); (d) the daily time in the using of board games (half hour, 1 hour, 2–3 hour, more than 3 hours). In **Table 2** was reported the core of Self-report Questionnarie. The scoring of intensity of use of digital and/or boards games was based on four-Likert-point scale (Item 2 and Item 4 of the questionnaire). The other questions applied a qualitative scoring.

**Table 2:** Self-report questionnaire.

(1) Which electronical device your child use autonomously or needs your help?	
Autonomously	Adult help
□ PC/laptop	□ PC/laptop
□ Ipad	□ Ipad
□ Wii/Xbox	□ Wii/Xbox
□ PSP	□ PSP
□ Mobile	□ Mobile
□ Other—————	□ Other—————
(2) During the day, how long time your child spends for electronical devices?	
□ Half hour	
□ 1 hour	
□ 2–3 hour	
□ More than 3 hours	
(3) Which board games your child use?	
□ Monopoli	□ Goose game
□ Risiko	□ Puzzle
□ Chess	□ Card’s play
□ Lady’s play	□ Other—————
□ Domino	
□ Scarabeo	
(4) How much time your child spends for board games during the week?	
□ Half hour	
□ 1 hour	
□ 2–3 hour	
□ More than 3 hours	
(5) Does your childlike to read books?	
□ Yes	
□ No	
□ Sometime	
□ Only if the parent reads it	
(6) If yes, when does he/she prefer to read:	
□ Afternoon	
□ Evening (to the bed)	
□ Only when the teacher asks it	
□ Other,—————	
(7) How long time your child spends for outdoor games (i.e., “blind fly game,” “hide-and-seek game,” “jump rope play,” “game with color command,” “1, 2, 3, stair game,” “bell game,” “football,” “volley,” “basketball” etc.)?	
□ Half hour	
□ 1 hour	
□ 2–3 hour	
□ More than 3 hours	

Applying questionnaire, we could classify the sample into two groups: (a) High Digital group (HD) = children that spend most high time for digital games; (b) Low Digital group (LD) = children that have spend most high time for board games.

### Ethics Statement

This study was carried out in accordance with the recommendations and approval of ‘Consiglio Didattico’ of the involved Schools with written informed consent from all subjects. All subjects gave written informed consent in accordance with the Declaration of Helsinki.

### Procedure

Testing was conducted into school attended by children, during school time. Participants have been tested in a quiet room reserved for testing. The test administration time was approximately 40 min. The examiners were trained Psychologists, graduate students from the Postgraduate School of Clinical Psychology of the University of L’Aquila, Italy. The examiners were blinded to the research objectives.

The procedure was composed of two detecting data steps: (1) by self-report parents and (2) children cognitive evaluation. First step was represented by the involvement of the parents to fill out the self-report questionnaire in order to detect the technology using form children in their daily time. Second step was the cognitive assessment of the children by application of psychological battery.

#### Self-Report Parents

Applying the self-report questionnaire it was possible to detect the frequency of the use of technology by children; the children were classified in High Digital users (HD) and Low Digital (LD) users.

#### Children Cognitive Evaluation

Standardized psychological tests have been applied to measure the cognitive abilities by quantitative data.

## Results

The data have been analyzed by the SPSS program version 20, and the significance level have been fixed at alpha < 0.05.

In **Table 3** are reported the raw scores of the sample in the psychological battery.

**Table 3:** Raw score of cognitive performance of sample in verbal and visuoperceptual distributed in four groups of age: 7, 8, 9, 10 year olds.

Tests		Sample (n. 191)		7 year olds group (n. 50)		8 year olds group (n. 49)		9 year olds group (n. 51)		10 year olds group (n. 41)		ANOVA	
		*M*	*SD*	*M*	*SD*	*M*	*SD*	*M*	*SD*	*M*	*SD*	*P*	η^2^
Visuoperceptual Tasks	Tower of London	9.69	1.7	9.14	2.0	9.45	1.8	10.0	1.4	10.2	1.4	0.008	0.83
	Corsi Tapping test	4.17	0.9	4.0	1.1	3.8	0.7	4.3	0.8	4.4	0.6	0.011	0.81
	Attentional Matrices	8.12	2.6	6.9	2.8	7.6	2.5	8.4	2.2	9.6	2.2	0.000	0.99
	Raven Colored Mat	24.69	5.5	22.6	7.5	24.4	4.7	25.5	3.8	26.3	4.3	0.006	0.86
Verbal Tasks	Naming	13.9	2.5	12.7	2.6	13.8	2.7	14.4	2.1	15.0	2.0	0.000	0.98
	Category Fluency	39.5	9.0	32.2	5.7	38.1	6.8	42.6	8.3	46.3	8.6	0.000	1.00
	Phonological Fluency	18.3	7.1	14.8	6.0	17.5	6.6	19.8	6.3	21.5	7.9	0.000	0.99
	Syntactic Comprehension	15.1	2.0	14.1	2.2	15.5	1.5	15.6	1.4	15.2	2.3	0.000	0.97

Firstly, data were elaborated by age variable. The sample was distributed into four groups of age: 7 year olds, 8 year olds, 9 year olds, 10 year olds groups. One-way-ANOVA was conducted to elaborate the performance of children along the age. As expected, children improve progressively their performance in verbal and visuoperceptual abilities: the results have evidenced significative effects for the groups in observed cognitive abilities (see **Table 3**).

Then, the scores obtained in standardized tests were transformed in *z* score and then we combined tests measuring visuoperceptual and verbal abilities.

Then, we divided the sample in n. 2 categorical digital use groups: high digital user (HD) and low digital user (LD); the applied criteria was the frequency and intensity of technology the children by detected data by experimental questionnaire filled out from parents. The distribution, as expected and inline of literature, resulted significant different: HD group was composed of n. 141 children (74%), and LD group n. 51 (26%).

In **Table 4** are reported the cognitive performance in z score.

**Table 4:** *z* score of the sample’s cognitive performance.

		Sample n. 191			
Tests		*z* score (HD n. 141; 74%)		*z* score (LD n. 51; 26%)	
		*M*	*SD*	*M*	*SD*
Visuoperceptual Tasks	Tower of London	0.03	1.0	–0.9	1.0
	Corsi Tapping test	0.09	1.0	–0.2	1.0
	Attentional Matrices	0.08	0.9	–02	0.9
	Raven Colored Mat	0.1	0.9	–0.3	1.0
Verbal Tasks	Naming	0.09	1.0	–0.2	0.9
	Category Fluency	0.1	1.0	–0.3	0.8
	Phonological Fluency	0.06	1.0	–0.1	0.9
	Syntactic Comprehension	0.03	1.0	–0.2	0.9

A multivariate analysis of variance (MANOVA) was conducted to determine the cognitive profile of sample distributed in two groups (HD and LD users) obtained in the composite score.

A MANOVA 4x2 on verbal tasks was conducted; the results have evidenced a significative multivariate effect for the groups [Pillai’s Trace *F*(4,186) = 0.690; *p* = 0.02; $\eta_{p}^{2}$= 0.05]; additionally, the Box’s M value of 13.2 was associated with a *p*-value of 0.23. Univariate results showed a significative effect for some verbal tasks: naming test [*F*(1,189) = 5.10; *p* = 0.02; $\eta_{p}^{2}$= 0.02] and Category fluency test [*F*(1,189) = 8.7; *p* = 0.004; $\eta_{p}^{2}$= 0.04].

**FIGURE 1:**
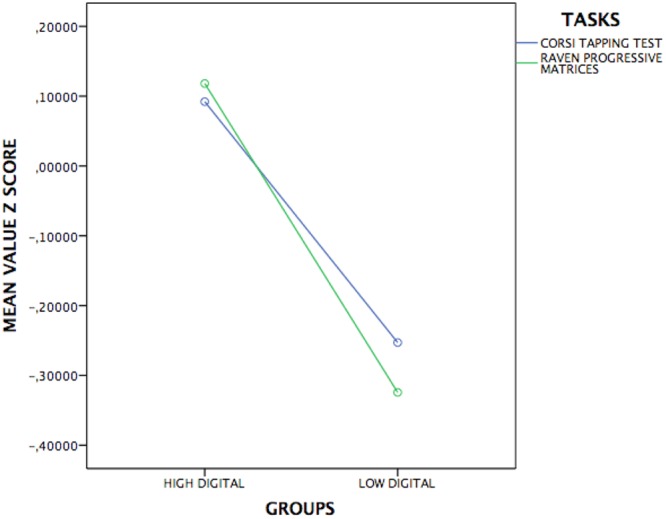
Representation of significant cognitive performance of HD and LD in visuoperceptual tasks.

**FIGURE 2:**
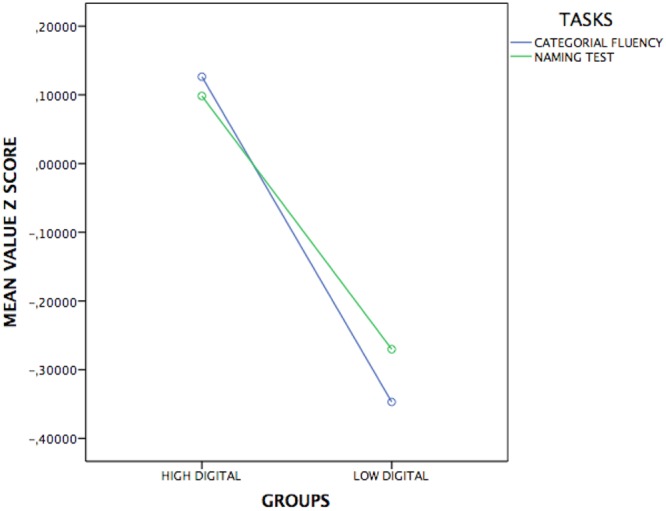
Representation of significative cognitive performance of HD and LD in verbal tasks.

Then, a MANOVA 4x2 was applied on visuoperceptual tasks and it has showed a significative multivariate effect [Pillai’s Trace *F*(4,186) = 2.7; *p* = 0.03; $\eta_{p}^{2}$= 0.05]; the Box’s M value of 16.8 was associated with a *p*-value of 0.09. Univariate showed a difference only on below visuoperceptual tasks: Corsi tapping test [*F*(1,189) = 4.5; *p* = 0.03; $\eta_{p}^{2}$= 0.02] and Raven Progressive Matrices [*F*(1,189) = 7.5; *p* = 0.007; $\eta_{p}^{2}$= 0.03]. In **Figures 1, 2** are represented the significative performance of two digital groups.

Finally, it has been examined the gender effect on two digital groups. A MANOVA 4x2x2 (4 tasks × 2 digital groups × 2 sex) on both verbal and visuoperceptual performance; it have evidenced no significative multivariate effects.

## Discussion

Aim of the research was to detect the impact of the technology in childhood in order to analyze its influence on cognitive development. Frequently, it is accepted the digital skills as enhanced learning: scholastic successful, intellective performance and daily advantage represent consistent variables in childhood and youth. Hsin et al. (2014) have highlighted two aspects in the development of digital skills: (a) skills and competences needed to use technology and (b) perceptions of technology use. Both of them are basic for the development of digital learning. Emerging debate is the measuring of the technology effectiveness.

Indeed, our results showed that the high digital skills are related to the improvement of the cognitive development. Children with high level have better performance in cognitive ability of language (naming and semantic skills) and visual (planning of the logical reasoning and visual memory). Those results are interesting: the digital exposition by gaming in early time could be a great instrument to favor the increased cognitive development of the children in digitalization world. Children so well-stimulated in early time by tasks related to pleasure (digital games) present an advantage in the intellective competence that will be useful the learning processing and much more in the adaptation to the educational and then working requests. Our data evidenced also a large technology use in childhood: the large part of children is digital skilled and can access to the technology in different way and easily; moreover, a small but relevant children group is digital unskilled and represents a lack of efficiency and equality to the access to the complexity of learning system.

In literature, our study is interesting because propose to investigate the efficacy and the functionally of the digital exposition in early age; more researchers have studied on the use of the technology in life of the people, in lifespan, but they were focused on quality of life. The key point of our study is the evidence that the digital skills are featured by tailored cognitive performance. Our research wanted to contribute to the new research trend of the digital impact on the cognitive development of human like technology is not only the enhancing of the learning processing but also just a good instrument for the intellective development. Technology has a strong impact on people life not only in terms of changing but much more of better leaping up the individual potentiality.

Our finding confirms the data present in literature and suggests the strong impact of the technology using not only in the social, educational and quality of life of the people, but also it outlines the functionality and the effect of the digital exposition in early age in order to increase the cognitive abilities of the children tailoring digital skilled generation with enhanced cognitive processing.

Secondary data of our study was the detection of a small (definitely) but still present group of children technologically unskilled, just because inaccessible in early time of life. That is a relevant aspect representing the early disadvantage (external variable) to develop the smart learning following traditional educational scheme.

## Conclusion

The technology has a strong impact on the quality of life of the human: the exposition of the children in young age to digital gaming favors advantage of learning capability not only in terms of high successful in educational path, but much more in the cognitive functionality by enhancing the verbal/visuoperceptual performance. The technology changes the life of the people improving the wellness in the social context and refining the learning processing, enhancing the cognitive performance and so favoring high adaptability in the elaboration information processing. Taking into account that, the learning processing reflects the changing becoming ‘smart learning’ for improved cognition.

A limit of present study is to have detected the digital skills by self-report method; beside we applied a standard measurement of cognitive performance. Could be interesting to measure digital skills by direct measurements and evaluate the correlated effect on cognitive development in childhood. Moreover, our preliminary analyses are interesting and significant to outline our next research focus for future investigations. Implications of our study can be in future leaning context and procedures in order to support educational needs of new generations fitting the efficacy on the cognitive potentiality.

## Author Contributions

The study was leaded by DG; and she wrote and analyzed data. JR has collaborated to the data collecting. PL has supervised the manuscript.

## Conflict of Interest Statement

The authors declare that the research was conducted in the absence of any commercial or financial relationships that could be construed as a potential conflict of interest.
